# Complete Genome Sequencing of *Borrelia valaisiana* and *Borrelia afzelii* Isolated from *Ixodes persulcatus* Ticks in Western Siberia

**DOI:** 10.1128/genomeA.01315-14

**Published:** 2014-12-24

**Authors:** Alexander M. Kurilshikov, Nataliya V. Fomenko, Oleg V. Stronin, Artem Y. Tikunov, Marsel R. Kabilov, Alexei E. Tupikin, Nina V. Tikunova

**Affiliations:** a Institute of Chemical Biology and Fundamental Medicine SB RAS, Novosibirsk, Russia; b Virion Scientific Production Association (NPO Microgen), Tomsk, Russia; c JSC Vector-Best, Novosibirsk, Russia

## Abstract

Lyme disease, caused by bacteria of the *Borrelia burgdorferi sensu lato* complex, is the most frequent tick-borne infection in Eurasia. Here, we report the complete genome sequence of the *Borrelia valaisiana* Tom 4006 and *Borrelia afzelii* Tom 3107 strains isolated from *Ixodes persulcatus* ticks in western Siberia.

## GENOME ANNOUNCEMENT

Lyme disease, caused by bacteria of the *Borrelia burgdorferi sensu lato* complex, is the most frequent tick-borne infection in Eurasia and North America. In Russia, *Borrelia afzelii* and *Borrelia garinii*, which belong to the *B. burgdorferi sensu lato* complex, are the most important causative agents of this disease (1). These bacteria are transmitted by *Ixodes persulcatus* ticks and approximately 50% of the ticks are infected with these bacterial species in western Siberia (2). Despite the common vector, both *B. garinii* and *B. afzelii* can prevail in different regions of Russia. *B. garinii* is mainly detected in western Siberia. Recently, *Borrelia valaisiana*, also associated with Lyme disease and previously found only in *Ixodes ricinus, Ixodes nipponensis*, and *Ixodes granulatus* ticks, was identified in *I. persulcatus* ticks collected in western Siberia and in a patient bitten by a *I. persulcatus* tick in far east Russia (3–5). The ability of *B. valaisiana* to be transmitted by different *Ixodes* species could require genetic variations to ensure its adaptation to the specific reservoir host.

Currently, more than 20 complete genomes of bacteria from the *B. burgdorferi sensu lato* complex have been sequenced, and only 3 of them belong to *B. garinii* and *B. afzelii* strains isolated from *I. persulcatus* (2 and 1, respectively) (6). There is one complete genome sequence for the *B. valaisiana* strain isolated from *I. ricinus* (7) and none for *B. valaisiana* isolated from *I. persulcatus*. Here we report the first complete genome sequence of the *B. valaisiana* Tom 4006 strain isolated from *I. persulcatus* ticks. In addition, we sequenced the complete genome of the *B. afzelii* Tom 3107 strain isolated from *I. persulcatus*.

The Tom3107 and Tom4006 strains were isolated from unfed *I. persulcatus* females collected by flagging vegetation in pine forests in the Tomsk region. *Borrelia* cells from the fourth passage on BSK-H medium were used to avoid possible plasmid diminution.

Bacteria were sequenced on an Illumina Miseq platform using paired-end sequencing. The length of the tag used was 150. Genomes were assembled by CLC Genomics Workbench software 6.0.0 with more than 200× coverage. The lengths of the master chromosomes were 905,861 and 912,160 bp for strains Tom 3107 and Tom 4006, respectively. Sequencing was performed in the SB RAS Genomics Core Facility, Novosibirsk, Russia.

### Nucleotide sequence accession numbers.

Master chromosomes of sequenced bacteria as well as cp26 and lp54 plasmids were uploaded to GenBank under accession no. CP009212, CP009213, and CP009214 (Tom3107) and CP009117, CP009118, and CP009119 (Tom4006).
